# Prognostic Potential of the NRF2 Transcription Factor in Canine Mammary Neoplasms

**DOI:** 10.3390/cancers18071107

**Published:** 2026-03-29

**Authors:** Vitor de Moraes Pina de Carvalho, Anna Hielm-Björkman, Karine Araújo Damasceno, Thanielle Novaes Fontes, Carlos Humberto da Costa Vieira-Filho, Simone Nunes, Diego Carlos dos Reis, Robin Moore, Stella Maria Barrouin-Melo, Geovanni Dantas Cassali, Alessandra Estrela-Lima

**Affiliations:** 1 Research Center on Mammary Oncology (NPqOM/HOSPMEV), Federal University of Bahia, Salvador 40170-110, Bahia, Brazil; vitor.moraes@ufba.br (V.d.M.P.d.C.); karine.damasceno@fiocruz.br (K.A.D.); thanielle.novaes@ufba.br (T.N.F.); chdfilho@ufba.br (C.H.d.C.V.-F.); simone.nunes@fiocruz.br (S.N.); barrouin@ufba.br (S.M.B.-M.); 2 DogRisk Research Group, Department of Equine and Small Animal Medicine, Faculty of Veterinary Medicine, University of Helsinki, 00790 Helsinki, Finland; anna.hielm-bjorkman@helsinki.fi (A.H.-B.); robin.moore@helsinki.fi (R.M.); 3 Laboratory for Global Health Research and Neglected Diseases, Gonçalo Moniz Institute, Oswaldo Cruz Foundation, Salvador 40296-710, Bahia, Brazil; 4 Division of Molecular Pathology, The Institute of Cancer Research, London SM2 5NG, UK; diego.dosreis@icr.ac.uk; 5 Department of Anatomy, Pathology, and Veterinary Clinics, Federal University of Bahia, Salvador 40170-110, Bahia, Brazil; 6 Laboratory of Comparative Pathology, Department of General Pathology, Federal University of Minas Gerais, Belo Horizonte 31270-901, Minas Gerais, Brazil; cassalig@icb.ufmg.br

**Keywords:** dog, biomarker, breast cancer, H-score, oxidative stress, transcription factor

## Abstract

Research based on the similarities between canine and human mammary tumors should extend beyond clinical, pathophysiological, epidemiological, and histopathological characteristics to include applicable molecular markers with prognostic significance. However, despite shared similarities, important differences must also be considered in comparative and translational studies. The nuclear erythroid 2-related factor (NRF2), a nuclear transcription factor that regulates the expression of antioxidant proteins, is pathologically activated during carcinogenesis. The role of NRF2 in human breast cancer is well established, making it a potential prognostic marker. **Objectives**: This study aimed to evaluate NRF2 tissue expression in mammary neoplasms of female dogs and its association with tumor progression, other prognostic factors, and survival. **Methods**: A group of 57 female dogs was studied. Tissue samples of mammary glands from 10 healthy dogs and 47 dogs with mammary neoplasms (39 malignant tumors and 8 benign tumors) were examined for NRF2 immunoexpression. Clinicopathological data and immunohistochemical expression, assessed by histochemical score (H-score), were correlated. **Results**: NRF2 tissue expression showed a predominantly cytoplasmic distribution and a lower H-score in tumors with higher malignancy grading. Dogs with higher NRF2 H-scores had improved survival rates (*p* = 0.0036). Univariate analysis revealed significant associations between H-scores < 135 and behavior (*p* = 0.007), tumor size (*p* = 0.001), and Ki-67 index (*p* = 0.018). **Conclusions**: These results suggest that NRF2 also holds prognostic value in the evaluation of canine mammary tumors.

## Simple Summary

Research shows that mammary tumors in dogs and humans share similarities, suggesting they may have common biological markers that predict disease progression. Despite these similarities, the two species differ significantly in malignancy rates and histological type. An important factor in tumor development is the protein NRF2, which helps manage cellular oxidative stress. Our study analyzed NRF2 in canine mammary tumors compared to normal tissue. In more aggressive tumors, NRF2 expression was lower and was associated with shorter survival. NRF2 levels were also associated with tumor behavior, size, and cell division rate. These findings suggest that NRF2 could be a valuable tool for predicting survival outcomes in dogs with mammary tumors.

## 1. Introduction

Breast cancer is the most prevalent neoplasm among women worldwide [1]. A detailed understanding of the development and progression of mammary tumors is essential for improving diagnostic methods and therapeutic strategies. In this context, mammary tumors in female dogs provide a unique opportunity for study, as they share significant biological and genetic characteristics with human breast cancer. This similarity establishes canine mammary tumors as valuable models for investigating tumor biology and potential treatments [2,3,4,5,6]. Identifying specific biomarkers capable of predicting disease outcomes is essential, as human and canine mammary tumors may differ significantly in their progression [2,7,8,9,10,11].

The nuclear erythroid 2-related factor (NRF2) regulates the expression of genes involved in oxidative stress responses, inflammation, and cellular metabolism [12]. Under physiological conditions, NRF2 is present in the cytoplasm at very low levels, bound to the Kelch-like ECH-associated protein 1 (KEAP1) [13]. KEAP1 forms a complex with Cullin 3 (Cul3) and RING box protein 1 (Rbx1), which ubiquitinates NRF2, leading to its proteasomal degradation. Under oxidative stress, NRF2 dissociates from KEAP1 and translocates to the nucleus, where it forms heterodimers with small Maf (musculoaponeurotic fibrosarcoma) proteins and activates antioxidant and detoxification response genes [14,15]. The NRF2-KEAP1 pathway is essential for regulating the cellular redox balance.

Owing to the interactions among reactive oxygen species (ROS) pathways, NRF2 signaling, and carcinogenesis, NRF2 modulation is considered an important target in cancer therapy [16,17], and its analysis has been proposed as a prognostic factor in breast tumors in women [18,19,20,21]. NRF2 activation has beneficial functions in cancer prevention, including eliminating carcinogens, ROS, and other DNA-damaging agents, thereby protecting normal cells from oxidative damage and inflammation [12]. However, NRF2 may also adopt a dual role in cancer [22]. In established tumors, continuous NRF2 activation may help neoplastic cells withstand high levels of endogenous ROS, evade apoptosis, promote tumor growth, and confer resistance to chemotherapy treatments [23,24].

In breast tumors in women, the expression of NRF2 occurs in the cell nucleus and is associated with higher aggressiveness and poorer prognosis [18,19,20,21]. However, NRF2 expression in mammary neoplasms of female dogs remains unknown. To the authors’ knowledge, only one study has reported NRF2 expression in canine osteosarcoma, with 100% of cases showing expression, yet with varying cellular localization and staining intensity among patients [25].

In this context, this study aimed to evaluate NRF2 expression in spontaneous mammary tumors of female dogs and its association with tumor biological behavior and malignancy grade. By assessing its correlation with classical prognostic factors (clinical staging, tumor size, and histopathological grading), the study evaluated its potential to serve as an independent prognostic factor for survival.

## 2. Materials and Methods

### 2.1. Ethical Approval

The Ethics Committee for the Use of Experimental Animals of the School of Veterinary Medicine and Zootechny of the Federal University of Bahia approved this research project (permit number 61/2022, approved on 4 October 2023). All the procedures followed the Brazilian College of Animal Experimentation (COBEA).

### 2.2. Study Design and Tumor Samples

**Figure 1:**
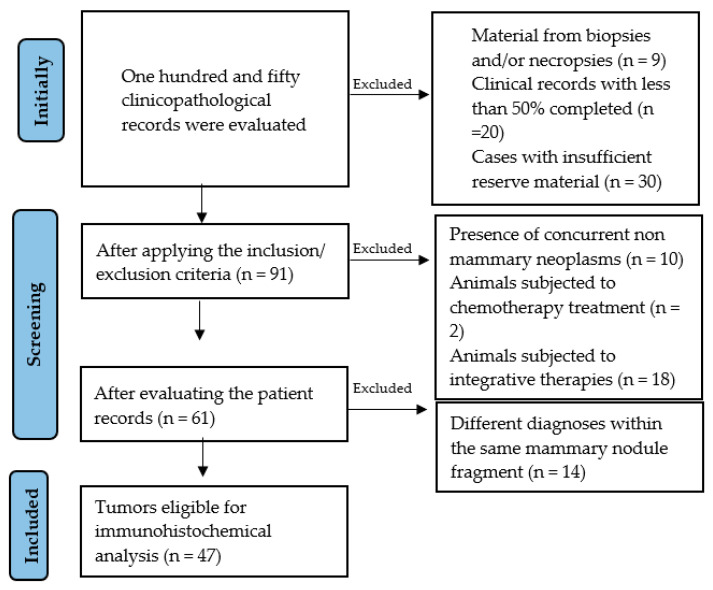
Flowchart for the selection of study cases applying the inclusion and exclusion criteria.

In this retrospective study, 150 clinicopathological records of female dogs of various breeds and ages diagnosed with mammary neoplasms between 2021 and 2023 were evaluated at the Mammary Oncology Research Center of the Federal University of Bahia, Brazil. Of the 150 records, 47 cases were selected for the study group according to the inclusion and exclusion criteria outlined in Figure 1. Recruited cases satisfied the following inclusion criteria: (a) available medical records with clinical and pathological information about tumor size, clinical staging, diagnosis, histopathological grade, and survival time; (b) biopsy collected from mastectomy; and (c) sufficient reserve samples for histological reprocessing and immunohistochemical analysis. In cases of multicentric tumors, the tumor with the worst prognosis (higher grade and most aggressive histological type) was selected.

The exclusion criteria applied were: cases with examined and reserve samples obtained from necropsy; cases with different histopathological diagnoses within the same mammary nodule fragment (in cases with multiple tumors); and cases with other concomitant non-mammary neoplasms. Dogs that had undergone neoadjuvant chemotherapy or integrative therapies (ozone therapy or medical cannabis) were also excluded.

The data obtained from the clinical records included age, breed, spaying status, histological grade and type, tumor size, nodal or distant metastasis, clinical staging, and survival status at the end of the study. The overall survival time, expressed in days, was defined as the time between the surgical excision of the primary tumor and the date of death from any cause or the end of the study (May 2023). The cutoff points for short (≤365 days) and long (>365 days) survival were used as recommended by Diniz-Gonçalves et al. (2023) [26].

Three study groups were established based on histological characteristics of the mammary glands. A control group (*n* = 10) comprised cases whose mammary parenchyma samples had no histological changes, that died due to traumatic causes, and that were examined at the Veterinary Pathology Laboratory; these animals had no previous history of mammary pathologies. A group of benign tumor cases (*n* = 8) consisted of mixed benign tumors (*n* = 6) and simple adenomas (*n* = 2). The group of malignant tumor cases (*n* = 39) consisted of simple carcinomas (*n* = 10), carcinosarcomas (*n* = 8), and mixed tumor carcinomas (*n* = 21), with this last group further subdivided into grade I (*n* = 11) and grade II−III (*n* = 10).

### 2.3. Histological Classification and Grading

Two pathologists independently and blindly examined the original hematoxylin-eosin (HE) slides for each case, without knowledge of the prior diagnosis. When necessary, new histological sections were prepared from the original paraffin blocks and stained with HE. The histopathological classifications followed the criteria described in the literature [27,28]. Tumor histopathological grading was carried out using the Nottingham System [29], which evaluates the percentage of tubule formation, nuclear pleomorphism, and the mitotic index. Canine mammary tumors (CMTs) were classified into Grades I, II, or III based on invasiveness [30]. Any discrepancies were resolved by discussion of multiheaded microscopy results to reach a consensus. Finally, cases with diagnoses and grades confirmed by both evaluators were included in the study. To assess agreement between the diagnoses of the two pathologists involved in the histopathological analysis and the assignment of NRF2 H-scores, the Kappa concordance coefficient was calculated, yielding a value of 0.90.

### 2.4. Immunohistochemistry

Expression of the NRF2 and Ki-67 markers was evaluated using immunohistochemical analysis on 4 μm sections from representative blocks of each mammary tumor sample (fragment). Tumor sections were deparaffinized in xylene and progressively hydrated in alcohol. Next, they were treated with sodium citrate buffer (pH 6.0) under moist, pressurized conditions at 115 °C for 20 min to expose the antigen of interest. Endogenous peroxidase activity was blocked with 10% hydrogen peroxide diluted in methanol, and nonspecific binding was blocked with Protein Block (Novolink Max Polymer Detection System) for 45 min. Subsequently, the slides were incubated overnight at 4 °C with the anti-NRF2 antibody (1:100, clone HL1021, ab313825, Abcam, Cambridge, UK) and the anti-Ki-67 antibody (1:100, clone MIB-1, Dako, Carpinteria, CA, USA). Following this, polymeric detection was performed according to the manufacturer’s instructions (Novolink Max Polymer Detection System). The sections were exposed to the chromogen 3,3′-diaminobenzidine (Novolink Max Polymer Detection System) and counterstained with Mayer’s hematoxylin. For the negative control, the primary antibodies were replaced with antibody diluent, and positive controls of the anti-NRF2 antibody were performed on kidney or mammary gland sections from healthy female dogs, following the manufacturer’s instructions.

To choose the anti-NRF2 antibody, the sequence homology between the target human and canine antigens was compared using the Basic Local Alignment Search Tool (BLAST, https://blast.ncbi.nlm.nih.gov/Blast.cgi; 14 January 2026). The results revealed a high homology between the proteins of the two species, with 89% identity (e-value: 0.0), and the antibody was therefore considered suitable for use.

### 2.5. Immunohistochemical Evaluation

Nuclear expression of Ki-67 was quantified using ImageJ (version 1.54d, National Institutes of Health, Bethesda, MD, USA) in neoplastic epithelial cells across all study groups. The thresholding method used was the “Otsu Thresholding” algorithm. Slide images were captured at 400× magnification under a microscope. These images were converted to grayscale and thresholded to distinguish positively marked nuclei from unmarked ones. Following thresholding, nuclei were automatically quantified using the “Analyze Particles” plugin in ImageJ. The particle size was set between 50 and 200 pixels to ensure only complete nuclei were counted, while circularity was adjusted between 0.5 and 1.0 to exclude artifacts and to include nuclei with expected shapes. Ten random fields were photographed for each case, excluding areas of necrosis or intense cell density, and 1000 neoplastic cell nuclei were quantified regardless of antibody marking. Areas of intense cell density were considered hypercellular, resulting from inflammation, dense aggregates of neoplastic cells, or processing artifacts that prevented correct, individual nuclear quantification during automated analysis in ImageJ. Positivity for Ki-67 staining was determined for cell nuclei exhibiting a diffuse nuclear staining pattern. The quantitative value of Ki-67 expression was defined as the percentage of positively stained cells among 1000 cells per section (400× magnification). To diagnose a high rate of cell proliferation, a cut-off of ≥14% of stained nuclei was adopted [26,31]. Ki-67 quantification was performed in 41 samples.

The immunohistochemical staining index for NRF2 was determined using the H-score system as described in the literature [32,33]. Initially, a pathologist established a scoring system and then scored each case, completing the evaluation of all cases within 2 weeks to ensure consistent interpretation. Subsequently, another pathologist evaluated the slides individually and subjectively classified staining intensities, determining the percentage of areas for each classification. Scores of 0, 1+, 2+, or 3+ (for none, weak, moderate, and strong staining, respectively) were assigned for the NRF2 antibody marking. In each case, the formula H-score = (1 x% % of 1+ cells) + (2 x% % of 2+ cells) + (3 x% % of 3+ cells) was used to calculate the cytoplasmic staining. Finally, the H-score for the sample was determined as the arithmetic mean of the values assigned by each pathologist, thereby ensuring a high degree of agreement.

### 2.6. Performance Indices of NRF2 Expression

The cut-off value for the NRF2 tissue expression that discriminated evolution to death or survival was determined by the receiver operating characteristic curve (ROC curve) [34]. Global accuracy was evaluated using the area under the ROC curve (AUC) [35]. The formulae used for the performance analysis calculated sensitivity (Sens, Equation (1)), specificity (Spec, Equation (2)), positive predictive value (PPV, Equation (3)), and negative predictive value (NPV, Equation (4)).

(1) $$Sensitivity\left(Sens\right)=\left[\frac{true\;positives}{\left(true\;positive\;samples+false\;negative\;samples\right)}\right]\times 100$$

(2) $$Specificity\;(Spec)=\left[\frac{true\;negatives}{\left(true\;negative\;samples+false\;positive\;samples\right)}\right]\times 100$$

(3) $$Positive\;predictive\;value\;(PPV)=\left[\frac{true\;positive\;samples}{total\;of\;positive\;samples}\right]\times 100$$

(4) $$Negative\;predictive\;value\;(NPV)=\left[\frac{true\;negative\;samples}{total\;of\;negative\;samples}\right]\times 100$$

### 2.7. Statistical Analysis

The Kolmogorov–Smirnov normality test was used to determine whether the data were normally distributed, to select parametric or nonparametric tests for data analysis. Fisher’s exact test was used to compare proportions between the study groups. The Kruskal–Wallis test followed by Dunn’s test was used to compare H-scores across groups. Bonferroni correction was applied to Dunn’s test for multiple comparisons. For continuous data, comparisons between groups were performed using the one-way ANOVA test followed by Tukey’s post hoc test. Binary logistic regression analysis and Spearman’s correlation were used to compare the H-score with evaluated clinical parameters in univariate and multivariate analyses, with parameters including behavior (malignant), age, tumor size (≥5 cm), presence of nodal and distant metastasis, clinical staging (III, IV, and V), grading (III and IV), Ki-67 (≥14%), neutering (no), and survival (days). The Kaplan–Meier analysis was used to construct the survival function, and the log-rank test served to compare the different groups (high and low H-score groups). The ROC curve was used to determine the cut-off point for the H-score, with a significance level of *p* < 0.05 and a 95% confidence interval for a two-tailed analysis. The performance analysis yielded an AUC of 0.738, indicating strong discrimination. All assumptions of the regression model were verified. Linearity was assessed using residual plots; independence of errors was tested with the Durbin-Watson test; homoscedasticity was evaluated through scatter plots; and the absence of multicollinearity was confirmed by calculating the variance inflation factor. The analyses were performed using SPSS 26.0 for Windows and GraphPad Prism v.8.0.2 (GraphPad, San Diego, CA, USA).

## 3. Results

### 3.1. Clinicopathological Characteristics

Twenty-one cases presented tumors larger than five centimeters, representing 44.7% (21/47) of the tumors studied. Inguinal mammary glands (55.3%; 26/47) were the most common anatomical sites affected by the tumors, regardless of laterality. Ulceration was present in 40.4% (19/47) of the studied CMTs.

In the group of malignant tumor cases, most of the dogs were characterized as clinical stage III (23.1%; 9/39) with tumors larger than 5 cm but without metastasis. Eight cases were classified as stage IV, with metastasis limited to lymph nodes, while six were stage V with distant metastasis. Histological grades I and II predominated (48.4%; 15/39) among the tumors amenable to grading (Table 1). Tumor-related fatalities accounted for 46.2% (18/47) among the study group deaths.

### 3.2. NRF2 Tissue Expression and H-Score Evaluation

**Figure 2:**
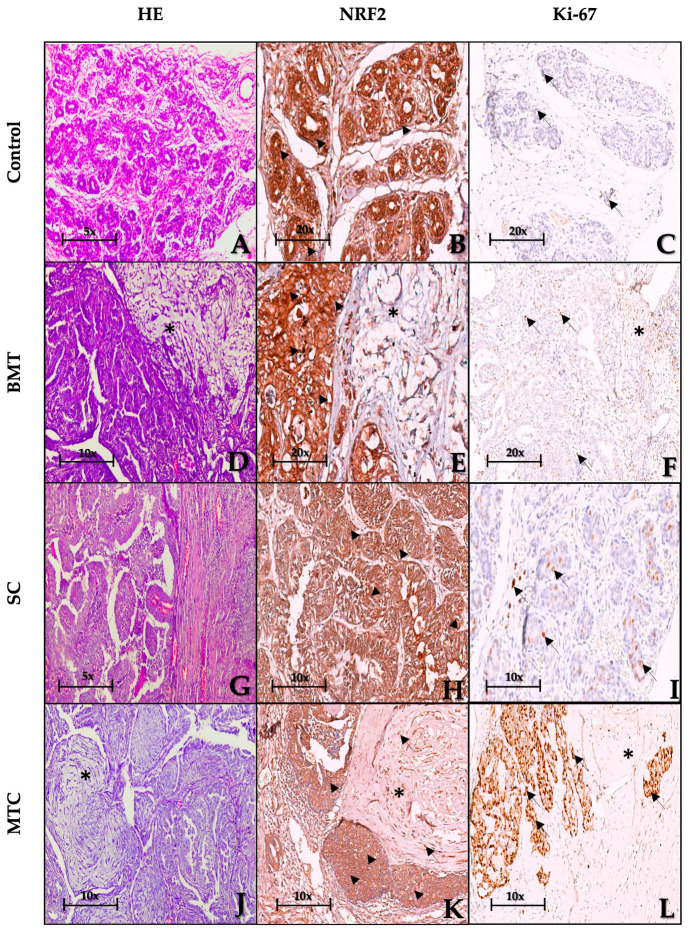
Histopathological images and immunohistochemical expression of NRF2 and Ki-67 markers in healthy mammary tissue (control dogs) and in mammary neoplasia samples of female dogs. (**A**) Mammary gland without neoplastic alteration under hematoxylin-eosin staining (HE). (**B**) Mammary gland without neoplastic alteration (NRF2). Intense cytoplasmic (arrowheads) and moderate nuclear (arrows) NRF2 expression. (**C**) Mammary gland without neoplastic alteration (Ki-67). Mild nuclear staining (arrows). (**D**) Benign mixed tumor (HE). (**E**) Benign mixed tumor (NRF2). Nuclear (arrows) and predominantly cytoplasmic (arrowhead) staining in benign epithelial cells. (**F**) Benign mixed tumor (Ki-67). Nuclear staining (arrows). (**G**) Simple carcinoma (HE). (**H)** Simple carcinoma (NRF2). Moderate and diffuse cytoplasmic staining in carcinomatous cells (arrowheads). (**I**) Simple carcinoma (Ki-67). Moderate nuclear staining (arrows). (**J**) Carcinoma in mixed tumor (HE). (**K**) Carcinoma in mixed tumor (NRF2). Predominantly weak cytoplasmic staining in carcinomatous cells (arrowheads). Myoepithelial cells with predominantly intense nuclear staining (arrows). (**L**) Carcinoma in mixed tumor (Ki-67). Moderate nuclear staining (arrows). (**B**,**C**,**E**,**F**,**H**,**I**,**K**,**L**) are counterstained with Mayer’s hematoxylin. (**A**,**C**,**K**,**L**) are viewed under a 5× objective, (**B**,**D**–**J**) under a 10× objective. *, myxoid matrix. BMT, benign mixed tumor. SC, simple carcinomas. MTC, carcinoma in mixed tumor.

The tissue expression of NRF2 was predominantly cytoplasmic and mostly diffuse in the tumor cells, while the staining intensity varied from absent (0+), through weak (1+) and moderate (2+), to strong (3+). In contrast, the normal mammary gland cells of the control group showed moderate-to-intense cytoplasmic marking, sometimes nuclear, and diffuse cytoplasmic staining. Interestingly, in the other two groups, only myoepithelial cells and a few epithelial cells showed nuclear NRF2 marking. Therefore, due to the scarce nuclear staining observed in the tumor cells in this study, only cytoplasmic staining was considered for the determination of the H-scores (Figure 2).

#### 3.2.1. NRF2 Expression in Canine Mammary Glands Without Neoplastic Alterations

In all samples of the control group, consisting of mammary parenchyma without histological alterations (Figure 2A), there was a predominant and diffuse cytoplasmic staining for NRF2 tissue expression (Figure 2B). Nuclear staining was moderate to intense and more frequent than in the other two groups. The mean H-score and standard deviation of the control group were 199 ± 45.8.

#### 3.2.2. NRF2 Expression in Canine Benign Mammary Neoplasms

Benign mammary neoplasms (8/47) exhibited intermediate H-scores for NRF2 tissue expression marking, with a mean and standard deviation of 183 ± 41.4. The expression was cytoplasmic and diffuse, but of lower intensity than in the control group (Figure 2E). Although the mean H-score was lower than that of the control group, this difference was not statistically significant. The benign neoplasms showed distinct H-scores when segregated by histological diagnosis. The benign mixed tumors (6/8) (Figure 2D) had a higher H-score (243.1 ± 104.4) for NRF2 expression than the simple adenomas (2/8) (191.5 ± 58.7).

#### 3.2.3. NRF2 Expression in Canine Malignant Mammary Tumors

The malignant tumor group (39/47) comprised five distinct histological types (Figure 2G,J). In general, NRF2 staining in this group was cytoplasmic, diffuse, and weak (Figure 2H,K), with a mean H-score of 115 ± 73.9. In a few tumors, mainly carcinosarcomas, there were areas with no staining (0). In most samples, NRF2 immunomarking was observed, but it was classified as weak (1+). However, among the simple carcinomas, NRF2 expression was moderate (2+) in most samples. The average H-scores and standard deviation for NRF2 expression in the simple carcinoma group were the highest (161.6 ± 61.7), followed by the grade I mixed tumor carcinomas (137 ± 61.9). The average NRF2 expression H-score for the more aggressive tumors, such as carcinosarcoma, was 35.6 ± 26.5. Grade II mixed tumor carcinomas had the lowest H-score averages (108.2 ± 80.3). There was only one sample of grade III mixed tumor carcinoma, whose H-score was 100.

#### 3.2.4. Comparative NRF2 Expression in Normal Mammary Gland vs. Benign and Malignant Mammary Neoplasms

**Figure 3:**
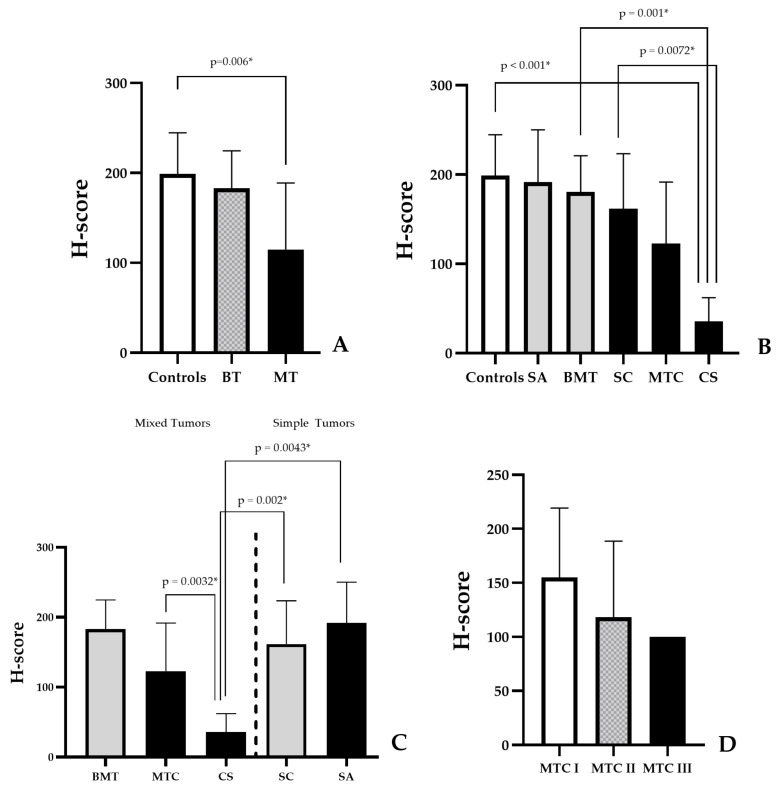
Graphical Representation of H-Score and Histological Types of Mammary Tumors in Dogs. (**A**) Comparison between the control group, benign tumors, and malignant tumors. BT, benign tumors. MT, malignant tumors. (**B**) Comparison between the malignant and benign versions of each tumor group. SA, simple adenomas. BMT, benign mixed tumors. SC, simple carcinomas. MTC, mixed tumor carcinomas. CS, carcinosarcomas. (**C**) Comparison between mixed tumors and simple tumors. (**D**) Comparison between different histological grades of mixed tumor carcinoma. MTC I, mixed tumor carcinoma I. MTC II, mixed tumor carcinoma II. MTC III, mixed tumor carcinoma III. * Significant differences (*p* < 0.05).

For the control, benign neoplasm, and malignant neoplasm groups, the H-scores were 199, 183, and 115, respectively. H-scores differed significantly across groups (H(2) = 12.55, *p* = 0.0019). The effect size was large (η^2^ = 0.195), indicating a substantial difference in NRF2 expression across groups. Post-hoc analysis showed a significant difference specifically between Control and Malignant groups (*p* = 0.006) (Figure 3A). It is noteworthy that H-scores decreased as tumors exhibited more malignant characteristics. The lower mean H-score in benign neoplasms compared with controls should be interpreted as a descriptive finding rather than a statistically significant difference.

#### 3.2.5. Comparative NRF2 Expression in Benign vs. Malignant Mammary Neoplasms

Lower NRF2 expression was observed in malignant breast tumor neoplasms compared to their benign counterparts. The statistically significant differences were primarily driven by the carcinosarcoma subgroup. Carcinosarcomas had lower H-scores than benign mixed tumors (*p* = 0.01). Mixed tumor carcinomas, regardless of grade, had lower H-scores than benign mixed tumors, but without statistical significance. However, for simple carcinomas of different grades (I and II) and histological types (papillary and tubular carcinoma), as well as simple adenomas, the differences in H-scores were not statistically significant.

There were significant differences in H-scores between the carcinosarcoma and the control (*p* < 0.001) and simple carcinoma groups (*p* = 0.0072) (Figure 3B).

#### 3.2.6. Comparative NRF2 Expression in Mixed vs. Simple Mammary Tumors

Comparing mixed tumors and simple tumors, a difference in NRF2 H-scores was observed by biological behavior, with lower expression as tumor aggressiveness increased. Benign mixed tumors showed a higher index than malignant mixed tumors, while carcinosarcomas exhibited lower NRF2 expression than mixed tumor carcinomas (*p* = 0.032).

Analysis of the simple tumors revealed a decrease in NRF2 expression with greater tumor aggressiveness, with simple adenomas showing higher NRF2 expression than simple carcinomas. However, these are only descriptive analyses, since there were only two simple adenoma samples.

The H-scores for the histological types of carcinosarcoma were significantly lower than those for simple carcinomas (*p* = 0.002) and simple adenomas (*p* = 0.043) (Figure 3C).

#### 3.2.7. Comparative NRF2 Expression in Mammary Carcinomas in Mixed Tumor Grades I vs. II and III

Regarding histological grading of carcinomas in mixed tumors, there was an inverse relationship between tumor grade and NRF2 H-scores. The higher the grade, the lower the tumor’s H-score (Figure 3D). These differences were not statistically significant and should therefore be interpreted with caution.

### 3.3. Expression of Ki-67 Versus NRF2

**Figure 4:**
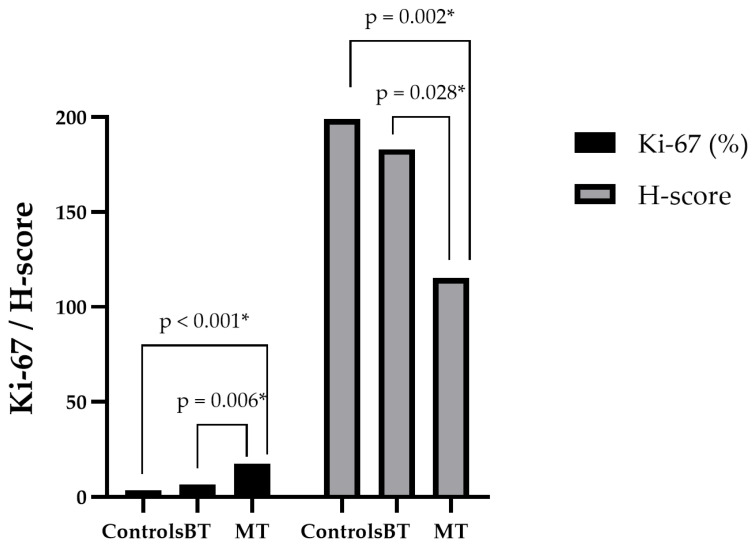
Comparison of Tumor Biological Behavior in Female Dogs and Average NRF2 and Ki-67 Expression. * Significant differences (*p* < 0.05).

Expression of the proliferative index marker Ki-67 was higher in malignant and aggressive tumors (Figure 2I,L) and significantly lower in benign mammary neoplasms (Figure 2F), followed by the control group (*p* < 0.001) (Figure 2C). The control group had a mean of 3.6 ± 1.3; benign tumors, 6.6 ± 1.2; and malignant tumors had a markedly higher mean of 17.4 ± 9.1. In the comparative analysis of Ki-67 expression versus NRF2 across all groups, an inverse relationship was observed: increased Ki-67 expression was associated with decreased NRF2 expression (Figure 4).

### 3.4. NRF2 Performance Index and H-Score Cut-Off

The proposed cut-off point for the NRF2 H-score was 135, defined as the value yielding the highest sensitivity (83.3%) and specificity (62.1%) while allowing for the detection of statistically significant differences and the investigation of associations with other variables.

**Figure 5:**
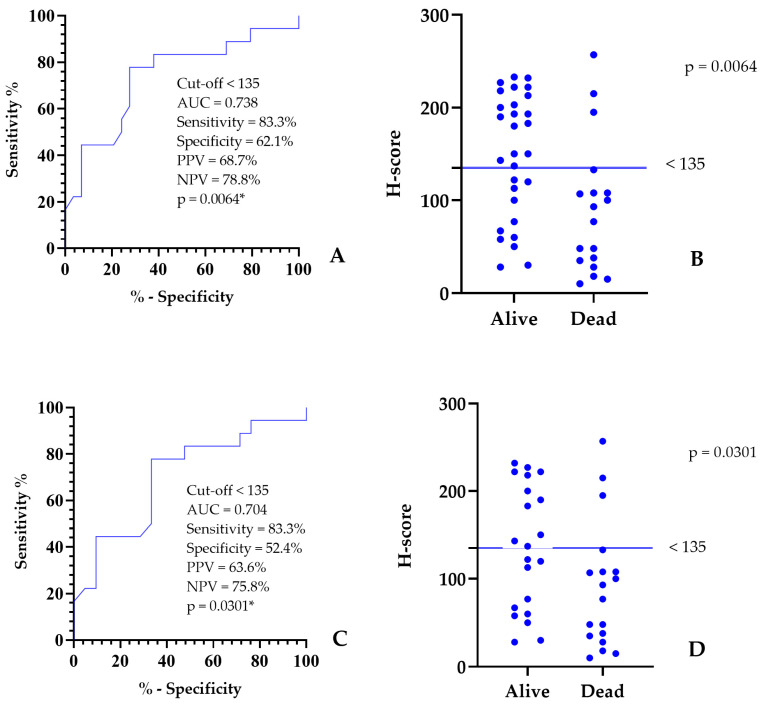
Graphical representation of the performance cut-off indices for NRF2 expression. (**A**) ROC (receiver operating characteristic) curve indices using a 365-day cut-off, including area under the curve/overall accuracy (AUC), sensitivity, specificity, positive and negative predictive values (PPV and NPV), and *p*-value. (**B**) Scatter plot of NRF2 expression versus disease outcome in female dogs with benign and malignant mammary tumors. Immunohistochemical quantification expressed by NRF2 to predict disease progression toward survival or death in female dogs with mammary neoplasia at 365 days. (**C**) ROC curve indices with malignant tumors only, including area under the curve/overall accuracy (AUC), sensitivity, specificity, positive and negative predictive values (PPV and NPV), and *p*-value. (**D**) Scatter plot of NRF2 expression versus disease outcome in female dogs with malignant mammary tumors. Immunohistochemical quantification expressed by NRF2 to predict disease progression toward survival or death in female dogs with mammary neoplasia at 365 days. * Significant differences (*p* < 0.05).

Performance analysis indices were strong, including an AUC of 0.738, indicating good discriminatory ability, a negative predictive value (NPV) of 78.8%, and a positive predictive value (PPV) of 68.7% (Figure 5A). Therefore, the NRF2 H-score serves as an effective predictor of mortality in this study (*p* = 0.0064). When evaluated over 365 days, cases with tumors having an NRF2 H-score ≥ 135 had long survival (>365 days), whereas those with an H-score < 135 had short survival.

Analysis of the scatter plot revealed that the H-score cut-off point for NRF2 expression (<135) identified statistically significant differences between mammary neoplasms and defined outcomes of disease progression toward survival or death in dogs. Survival analysis considered the alive/dead status of the canine patients with malignant and benign mammary neoplasms at 365 days post-mastectomy, 29 of whom survived, while 18 died (Figure 5B).

When analyzing only malignant mammary neoplasms without a 365-day cutoff, the ROC curve also indicated statistical significance, with an H-score cutoff of 135, a sensitivity of 83.3%, and a specificity of 52.4%. The performance analysis indices were also strong, with a global accuracy value (AUC) of 0.704, indicating good discriminatory capacity, an NPV of 75.8%, and a PPV of 63.6% (Figure 5C). This last analysis reinforces the value of the NRF2 H-score as a predictor of death in cases with malignant neoplasms in this study (*p* = 0.0301). Survival analysis considered the alive/death status of canine patients only with malignant mammary neoplasms after mastectomy, 21 of whom survived and 18 died (Figure 5D). To characterize the performance of the H-score cutoff point, its classification accuracy was 66.7% for malignant tumors alone and 75.2% for all tumors with a cutoff of 365 days.

### 3.5. Association of Clinicopathological Parameters with NRF2 Expression H-Score

Regarding the clinicopathological data, analysis revealed an association between the H-score for NRF2 expression and values for tumor behavior, tumor size, proliferative index (Ki-67), and survival. Malignant tumors had a 12.5-fold higher likelihood of presenting an H-score below 135 than benign tumors (OR = 12.5; 95% CI, 1.39–112.26). Similarly, tumors larger than five centimeters had an 8.94-fold higher chance of presenting an NRF2 H-score below 135 (OR = 8.94; 95% CI, 2.24–35.61). Cases with tumors that had a Ki-67 index ≥ 14 also had a 6.5-fold greater chance of having an NRF2 H-score below 135 (OR = 6.5; 95% CI, 1.28–33.04). Therefore, the larger the tumor size, the lower the NRF2 H-score. Regarding survival, the chance of canine patients dying because of their tumor disease when the tumor had an NRF2 H-score below 135 was 8.18-fold higher than for those with an H-score above 135 (OR = 8.18; 95% CI, 1.92–34.84) (Table 2).

### 3.6. Comparison of Survival Curves

**Figure 6:**
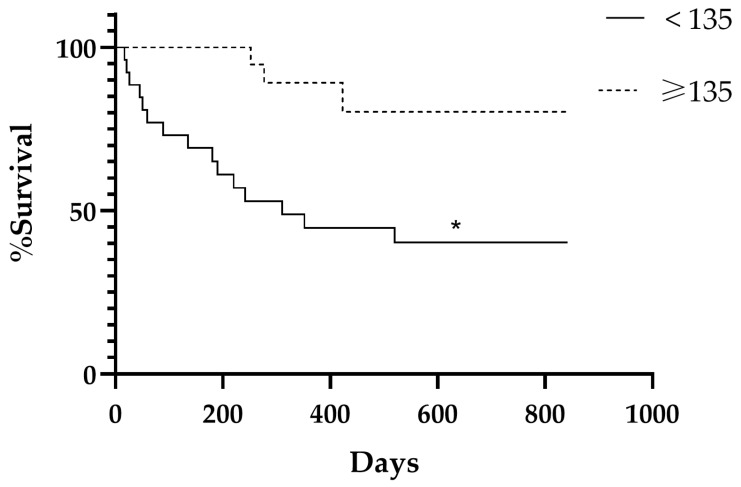
Prognostic impact of NRF2 H-score expression on overall survival in dogs with mammary tumors. Kaplan–Meier survival curve for all animals with mammary tumors, stratified by NRF2 H-score (low < 135 and high ≥ 135). * Expresses a significant statistical difference between the curves by the log-rank test with *p* = 0.0036.

Comparing the survival curves, NRF2 expression in neoplastic epithelial cells was considered high when the H-score was ≥135 and low when the H-score was <135. Stratification based on NRF2 expression indicated a significant difference in the survival curves for H-scores < 135 and ≥135 (*p* = 0.0036; HR = 0.19; 95% CI, 0.076–0.486). Dogs with tumors that exhibited lower NRF2 expression (H-score < 135) had a median survival of 311 days and a higher mortality rate (*n* = 15/47, 31.9%). The mortality rate in the group with tumors with an H-score ≥ 135 was lower (3/47, 6.4%) and did not reach the median survival rate (Figure 6). When the analysis was restricted to malignant mammary neoplasms, the association between NRF2 expression and survival remained statistically significant. Survival curves again revealed separation between the groups (*p* = 0.0018; HR = 0.25; 95% CI, 0.009–0.638), reinforcing the prognostic relevance of NRF2 expression in malignant tumors.

### 3.7. Univariate and Multivariate Data Analysis

Univariate data analysis revealed that malignant behavior (*p* = 0.007), tumor size ≥ 5 cm (*p* = 0.001), distant metastasis (*p* = 0.048), and a Ki-67 index ≥ 14% (*p* = 0.018) correlated with NRF2 expression (high ≥ 135 and low < 135). In the multivariate analysis, behavior (malignant), tumor size (≥5 cm), and Ki-67 index (≥14%) also significantly correlated with NRF2 expression, with odds ratios of 12.5 (95% CI, 1.39–112.26), 8.94 (95% CI, 2.24–35.61) and 6.5 (95% CI, 1.28–33.04), respectively. These findings support the applicability of the parameters ‘tumor size’ and ‘Ki-67 index’ as independent risk factors in the evaluation of NRF2 expression in canine mammary tumors (Table 3).

## 4. Discussion

The transcription factor NRF2 is an important prognostic marker in breast tumors in women [18,19,20,21]. Owing to their reported similarities, the female dog with spontaneous mammary tumors is an important comparative and translational model for the study of breast cancer in women [2,3,4,5,6]. However, to the authors’ knowledge, there are no literature reports of studies exploring NRF2 in canine mammary tumors.

In this context, this study is pioneering in evaluating NRF2 expression in mammary tumors of female dogs and its association with classical prognostic factors. Immunostaining methods used to identify and quantify NRF2 expression in mammary tumors of female dogs revealed a predominantly cytoplasmic, diffuse distribution. We also found that NRF2 expression was scarce or absent in the nuclei of neoplastic epithelial cells. For this reason, we focused on cytoplasmic NRF2 expression to proceed with evaluation and association with clinicopathological parameters, including tumor malignancy, histopathological classification, proliferation index (Ki-67), clinical staging, tumor size, and survival. A pattern in NRF2 expression enabled the establishment of tumor malignancy potential in dogs. While NRF2 expression staining was moderate-to-intense and diffuse in control samples, benign tumors, and well-differentiated carcinomas, in aggressive tumors, it was absent or less intense. In contrast, in a study on human breast cancer, the authors found a predominantly nuclear expression of NRF2 associated with higher histological grading and poorer prognosis [21]. However, a previous study investigating human head and neck tumors also described cytoplasmic localization of NRF2. These tumors were associated with a poor prognosis, harbored mutations in the KEAP1 and NRF2 genes, and showed high NRF2 immunohistochemical expression [36].

In the present cases, although cytoplasmic NRF2 expression was more pronounced in benign neoplasms than in malignant neoplasms, nuclear NRF2 expression remained minimal across the tumor types. Oxidative stress is widely recognized as a crucial factor in the prognosis and treatment response in both women [37,38,39,40,41] and female dogs [42] with mammary cancer. Oxidative stress triggers important signaling for the nuclear translocation of NRF2 [22]. The higher cytoplasmic NRF2 expression we observed in benign neoplasms might indicate that their cells have an enhanced ability to detect and respond to perturbations in the cellular redox environment compared to malignant cells. Indeed, neoplastic cells generally present high levels of ROS [43,44], but these oxidative stress signals may be transient.

It is also likely that the nuclear localization of NRF2 requires additional increases in ROS levels and may be influenced by other factors. To interpret and understand NRF2 cytoplasmic expression, it is important to consider certain factors, including the nuclear localization signals (NLS) and export signals (NES) within NRF2, the interaction between this transcription factor and importin proteins [45,46], mutations in NRF2 or KEAP1, and the involvement of the p53 and CRM1 genes [36,47,48,49,50]. Thus, the present results highlight the need to explore the functional significance of cytoplasmic NRF2 expression in mammary neoplasms of female dogs. Moreover, the nuclear-cytoplasmic transport mechanisms of this transcription factor should be investigated.

Although NRF2 is a nuclear transcription factor, it also occurs in the cytoplasm bound to KEAP1, and its cytoplasmic immunostaining is often positive [51]. This may explain the predominance of cytoplasmic staining observed in the present study. However, further studies are needed to evaluate the expression of proteins associated with NRF2 in mammary tumors. Lee et al. (2023) [19] studied NRF2 and KEAP1 expression in triple-negative breast tumors in women and found poorer survival among women with higher nuclear NRF2 expression and lower cytoplasmic KEAP1 expression. Thus, the role of KEAP1 as a prognostic factor in mammary tumors needs clarification.

Physiologically, the NRF2 bound to KEAP1 in the cytoplasm of cells is a marker of cellular oxidative stress and is sensitive to oxidative imbalances [13]. As reactive oxygen species (ROS) levels increase, NRF2 translocates to the nucleus, where it associates with small Maf proteins to activate target genes through the antioxidant response element (ARE) [15]. Studies have demonstrated a link between oxidative imbalance and malignant neoplasms [52,53]. In view of this, we expected to find higher nuclear staining in malignant tumors and more pronounced cytoplasmic staining in benign neoplasms, similar to findings reported on human breast tumors [18,19,20,21]. However, we hypothesize that the absent or reduced nuclear NRF2 expression observed in the epithelial cells of canine mammary tumors may result from complex dysregulation of signaling pathways in these neoplastic cells.

Human pleomorphic adenoma of the salivary gland and its malignant counterpart, the carcinoma ex pleomorphic adenoma, share several characteristics with benign and malignant mixed tumors of the canine mammary gland [54]. Droździk et al. (2015) [55] investigated the role of NRF2 in salivary pleomorphic adenomas and reported nuclear expression; they also identified cytoplasmic expression in neoplastic epithelial cells, confirming NRF2 cytoplasmic localization in mixed tumors.

NRF2 marking in myoepithelial cells has also been reported in human salivary gland pleomorphic adenomas, appearing intense, diffuse, and nuclear [56]. This pattern is similar to that observed in the present study. The myoepithelial component of benign mixed tumors and the carcinomas within these tumors have benign behavior [57]. Indeed, diffuse nuclear staining in the myoepithelial cells was also observed in the present benign epithelial cells and in the control group. Therefore, these findings may suggest that nuclear NRF2 expression in mixed mammary tumors of female dogs is associated with benign biological behavior.

Regarding the biological behavior of mammary tumors and the histological grade of mixed tumor carcinomas, our findings on immunohistochemical quantification of NRF2 expression confirm that malignant tumors exhibit lower H-scores than benign tumors. Moreover, among malignant tumors, higher-grade tumors correlate with lower H-scores. A reason for this might be that neoplastic cells have a less efficient antioxidant system than normal cells, as evidenced by their elevated ROS levels and hyperactivation of NRF2 [43,44]. Similar high NRF2 expression has been reported in polymorphic adenocarcinoma of the human salivary glands, a neoplasm with a good prognosis [58].

The association between favorable prognosis and high NRF2 expression, as observed in this study, has been described in pulmonary mucoepidermoid carcinoma in humans [59]. These authors reported that NRF2 expression was positively associated with tumor-suppressive microRNAs, such as miR-181a, miR-193b, and miR-424, and negatively associated with the oncogenic miR-378, as well as with the overexpression of the heme oxygenase 1 (HO-1) gene [59]. A positive correlation was also observed between these markers and breast tumors in women [18]. Therefore, molecular pathways of NRF2 action should be investigated as mediators of the biological behavior of mammary tumors in female dogs.

In mixed canine mammary tumors, NRF2 expression decreased as malignancy increased. Likewise, simple tumors such as canine mammary adenomas and carcinomas exhibited lower NRF2 expression with more malignancy and tumor progression. Nevertheless, it is important to highlight the predominance of mixed mammary tumors among the canine cases in the present study. Mixed mammary tumors are rare in women and exhibit a complex histological pattern composed of epithelial, myoepithelial, and mesenchymal components [57].

The lower NRF2 expression observed in malignant breast neoplasms should be interpreted with caution, as statistically significant differences were identified mainly in carcinosarcomas, which have the lowest H-scores in the present series. Thus, the apparent reduction in NRF2 expression in malignant tumors may have been predominantly influenced by this more aggressive histological subtype.

One can assume that NRF2 expression in mixed tumors may decisively influence the divergent results reported for mammary tumors in female dogs and women. In this context, to better understand NRF2 expression in mammary tumors of female dogs, there is a need for more detailed evaluation of neoplastic myoepithelial and mesenchymal cells. Similar to the findings of this study, reported variations in NRF2 expression have been associated with the histological subtype or arrangement of the same tumor. One such study of human salivary gland cystic carcinomas showed higher NRF2 expression in tubular tumors than in cribriform tumors [56]. In the present study, simple tumors presented higher H-scores than mixed tumors, reinforcing the hypothesis that the histological composition influences NRF2 expression in canine mammary neoplasms.

Studies on prognostic factors in breast cancer in women have reported that the use of semi-quantitative or qualitative methods may contribute to discrepant findings in the evaluation of NRF2 expression [19,20,21]. To minimize potential discrepancies, we set a cutoff of 135 to determine the NRF2 expression H-score. This applied ROC curve analysis based on sensitivity, specificity, and negative and positive predictive values, allowing coherent quantification of NRF2 expression in canine mammary tumors across diverse categories compared to healthy canine mammary glands.

Evaluation of the proliferation marker Ki-67 supports the finding that a lower index of NRF2 expression could be associated with higher malignancy in canine mammary tumors. We found a negative association between Ki-67 and NRF2 marking, indicating that lower NRF2 expression was characteristic of malignant tumors with a high proliferation rate. These findings could be explained by the results of Kadthur et al. (2011) [60], which showed that high nuclear Ki-67 expression was associated with lower differentiation, a less favorable histopathological classification, and greater metastatic potential in canine mammary neoplasia. However, other studies on breast tumors in women report a positive association between the markers Ki-67 and NRF2 [19,20], which differs from this study’s findings regarding the proliferative capacity and NRF2 expression in canine mammary tumors. The association between Ki-67 and lower H-scores suggests that reduced NRF2 expression is associated with more proliferative tumors, supporting the hypothesis that NRF2 influences cell proliferation, possibly by regulating oxidative stress. Similarly, the association with larger tumors suggests that low NRF2 expression may be associated with reduced antioxidant capacity, thereby promoting tumor growth.

The univariate analysis showed that malignant behavior, tumor size, distant metastasis, and proliferation index (Ki-67) were related to NRF2 expression in canine mammary tumors. NRF2 expression was lower in large tumors, suggesting a more aggressive biological behavior. In contrast, a direct association has been reported between staging and NRF2 expression in breast tumors in women [20,61]. This divergence between women and canine mammary tumors suggests that several aspects of the underlying mechanisms of tumor development in both species remain to be studied. The pattern revealed in this study, with malignancy, tumor size, and proliferation index (Ki-67) inversely associated with NRF2 expression, was confirmed by multivariate analysis. Serving as independent predictors of malignant biological behavior, both tumor size and proliferation index support the prognostic value of NRF2 marking in canine mammary neoplasms. Future investigations based on these findings may contribute to the development of more targeted therapeutic strategies for mammary neoplasia in dogs and women.

Because the survival curves were strongly associated with NRF2 H-scores in the ROC analysis, NRF2 expression could also predict mortality in female dogs with various mammary neoplasms. This indicates that tumors with a lower H-score reflect a greater potential for malignancy and are associated with a higher likelihood of fatal progression. Rutland et al. (2021) [25] suggested a direct correlation between NRF2 expression and survival in canine osteosarcomas. Similarly, Onodera et al. (2014) [20] found a strong correlation between NRF2 expression and prognostic factors, as well as an increase in disease-free survival in breast cancer. In the present study, the association between NRF2 expression and survival in female dogs suggests that NRF2 may be an independent prognostic factor for mammary neoplasms in the canine species.

We acknowledge certain limitations of our study, including the relatively small sample size, which resulted in wide confidence intervals for some associations, limited statistical precision, and some uncertainty in the estimated effect sizes, as well as the lack of quantification of myoepithelial cells in canine mammary neoplasms. We recognize the need for additional experimental assays to fully evaluate and validate the cross-reactivity of the anti-NRF2 monoclonal antibody with the canine protein. Although the antibody choice was based on consistent technical and biological criteria, including molecular conservation data and prior applicability, such evidence might not replace the direct demonstration of specificity across a broader range of canine mammary tissues and tumors. Therefore, future complementary studies that further strengthen the evidence of the antibody’s cross-reactivity in a larger canine population, including analyses of related transcription factors such as KEAP-1 and HO-1, should be conducted.

However, we have achieved our objective of characterizing NRF2 expression using the currently available reagents to assess it in canine mammary tumors and have demonstrated the importance of providing a basis for future investigations. We emphasize that our findings should be interpreted as initial, biologically relevant evidence of the potential prognostic role of NRF2 in canine mammary neoplasms, but not as definitive validation of the underlying molecular mechanisms.

## 5. Conclusions

This study characterized NRF2 tissue expression in canine mammary tumors, revealing a predominantly cytoplasmic localization and diffuse distribution in epithelial cells. Immunohistochemical methods showed that the NRF2 H-score was lower in malignant tumors than in benign tumors or normal canine mammary glands. In addition, NRF2 expression showed a statistically significant inverse association with prognostic factors, including tumor size and the Ki-67 proliferation index. A cut-off point was proposed for the quantitative evaluation of NRF2 marking, enabling the identification of dogs with a greater probability of survival and highlighting its significant clinical value. Thus, NRF2 is a potential independent prognostic factor in canine mammary neoplasms. Further investigations based on these preliminary findings will refine the treatment and prognosis of these neoplasms in dogs, opening new perspectives for comparative studies and for translational approaches to develop new therapeutic and prognostic tools in humans.

## Figures and Tables

**Table 1:** Evaluation of Epidemiological and Prognostic Factors in Canine Mammary Tumors.

Parameters	Benign Tumors *n* = 8	Malignant Tumors *n* = 39
Neutering		
Yes	2 (25%)	10 (25.6%)
No	6 (75%)	29 (74.4%)
Size		
<3 cm	8 (100%)	9 (23.1%)
3–5 cm	0	9 (23.1%)
>5 cm	0	21 (53.8%)
Lymph Node Metastasis ^1^		
N0	0	29 (74.4%)
N1	0	10 (25.6%)
Distant Metastasis ^2^		
M0	0	33 (84.6%)
M1	0	6 (15.4%)
Clinical Staging		
I	0	8 (20.5%)
II	0	8 (20.5%)
III	0	9 (23.1%)
IV	0	8 (20.5%)
V	0	6 (15.4%)
Grade		
I	0	15 (48.4%)
II	0	15 (48.4%)
III	0	1 (3.2%)
Survival (due to tumor disease)		
Alive	8 (100%)	21 (53.8%)
Dead	0	18 (46.2%)

^1^ N0, without nodal metastasis; N1, with nodal metastasis; ^2^ M0, without distant metastasis; M1, with distant metastasis.

**Table 2:** Association Between Clinicopathological Parameters and H-score Interval.

		H-Score			Odds Ratio (CI ^1^ 95%)
		<135	≥135	*p*-Value	
		*n* (%)	*n* (%)		
Tumor Behavior	Benign	1 (3.8%)	7 (33.3%)	0.024 *	12.5
	Malign	25 (96.2%)	14 (66.7%)		(1.39–112.26)
Age	<11 years	11 (42.3%)	10 (47.6%)	0.716	1.24
	≥11 years	15 (57.7%)	11 (52.4%)		(0.39–3.94)
KI-67	<14%	3 (25%)	13 (68.4%)	0.024 *	6.5
	≥14%	9 (75%)	6 (31.6%)		(1.28–33.04)
Tumor Size	<5 cm	4 (15.4%)	13 (61.9%)	0.002 *	8.94
	≥5 cm	22 (84.6%)	8 (38.1%)		(2.24–35.61)
Lymph Node Metastasis	N0	17 (68%)	12 (85.7%)	0.236	0.35
	N1	8 (32%)	2 (14.3%)		(0.06–1.97)
Distant Metastasis	M0	19 (76%)	14 (100%)	0.999	NA ^2^
	M1	6 (24%)	0		
Grade	I	6 (35.3%)	9 (64.3%)	0.113	3.3
	II and III	11 (64.7%)	5 (35.7%)		(0.75–14.45)
Clinical Staging	I and II	7 (28%)	6 (42.9%)	0.348	1.93
	III, IV, and V	18 (72%)	8 (57.1)		(0.49–7.6)
Neutering	Yes	4 (15.4%)	8 (38.1%)	0.084	3.38
	No	22 (84.6%)	13 (61.9%)		(0.85–13.48)
Survival	Alive	11 (42.3%)	18 (85.7%)	0.004 *	8.18
	Dead	15 (57.7%)	3 (14.3%)		(1.92–34.84)

^1^ CI, Confidence interval; ^2^ NA, not available, as it cannot be calculated; * Significant differences (*p* < 0.05).

**Table 3:** Univariate and Multivariate Analysis of Clinicopathological Factors Related to H-score Expression < 135.

	Univariate Analysis	Multivariate Analysis
	*p*-Value	Odds Ratio (CI ^1^ 95%)
Behavior (malignant)	0.007 *	12.5 (1.39–112.26)
Age	0.297	0.88 (0.69–1.12)
Tumor Size (≥5 cm)	0.001 *	8.94 (2.24–35.61)
Lymph Node Metastasis (N1)	0.235	0.35 (0.06–1.97)
Clinical Staging (III, IV, and V)	0.358	1.93 (0.49–7.6)
Grade (II and III)	0.115	3.3(0.75–14.45)
KI-67 (≥14%)	0.018 *	6.5(1.28–33.04)
Neutering (no)	0.079	3.38(0.85–13.48)
Survival (days)	0.210	1.01(0.99–1.03)

^1^ CI, Confidence interval; * Significant differences (*p* < 0.05).

## Data Availability

The raw data supporting this article’s conclusions are available upon reasonable request.
